# Recycled Nylon Fiber from Waste Fishing Nets as Reinforcement in Polymer Cement Mortar for the Repair of Corroded RC Beams

**DOI:** 10.3390/ma13194276

**Published:** 2020-09-25

**Authors:** Teeranai Srimahachota, Hiroshi Yokota, Yoshikazu Akira

**Affiliations:** 1 Graduate School of Engineering, Hokkaido University, Sapporo 001-0014, Japan; 2 Faculty of Engineering, Hokkaido University, Sapporo 001-0014, Japan; yokota@eng.hokudai.ac.jp; 3 Faculty of Engineering, Kagoshima University, Kagoshima 890-0065, Japan; akira@oce.kagoshima-u.ac.jp

**Keywords:** fiber reinforcing mortar, polymer cement mortar, recycled fiber, waste fishing nets

## Abstract

Waste fishing nets were utilized as recycled nylon (RN) short fiber to improve the mechanical properties of cement mortar. RN and manufactured polyethylene (PE) fibers were added to polymer cement mortar (PCM) as a reinforcement, and fiber-reinforced PCM was sprayed on the section of reinforced concrete (RC) beams. Normal RC beams and the upgraded RC beams were placed in the tidal zone for 14 months to induce rebar corrosion. Consequently, a repair operation took place by the removal of the concrete cover then spraying fiber-reinforced PCM. The tested RC beams were subjected to four-point flexural tests to study their load-carrying capacity. It was found that the fibers helped transfer stresses through cracks and distribute stresses by transforming a single wide crack into many small cracks. Overall, the experimental results showed that recycled nylon fibers from waste fishing nets have great potential to be used as a strengthening fiber in cementitious material.

## 1. Introduction

Marine ecosystems are deteriorating due to derelict fishing gear (DFG). Fishing gears including lines, ropes and fishing nets that were discarded or lost in the ocean become traps, leading to the deaths of many marine species. It was estimated that DFG causes the deaths of 380,000 marine animals annually, especially whales, sea lions and turtles [1]. It is estimated that 5.7% of fishing nets, 8.6% of traps and 29% of lines from global fishery industries were lost into the ocean in 2017 [2]. DFG becomes the most common material entangling marine life, as well as causing damage to coral reefs [3]. For example, more than 58,000 cases of entanglement by DFG were reported in Korean seas, causing economic losses over 90 million USD [4]. Therefore, finding suitable solutions for collecting and recycling those fishing nets is an urgent issue to mitigate environmental impact.

Fishing nets are usually made of strong and durable materials, such as nylon, polyamide (PA) and high-density polyethylene (HDPE), which are non-biodegradable and require a lot of resources and energy, as well as emitting greenhouse gases, for their recycling [5]. Waste fishing nets can be recycled into many products, such as textiles, clothes, footwear and accessories [6,7]. In the concrete engineering field, synthetic fibers have been widely used as reinforcement in cementitious materials. For instance, polypropylene (PP), polyvinyl alcohol (PVA) and polyethylene terephthalate (PET) fibers were found to improve the mechanical properties and durability of concrete, such as flexural capacity, flexural toughness, shrinkage resistance and freeze-thaw resistance [8,9,10,11,12,13,14,15,16,17,18], and adding fibers was found to have a significant effect on the crack bridging and the failure behavior [19,20,21]. Moreover, it was found that adding PP fibers improves sulfate resistance and alkali-silica reaction (ASR) expansions [22,23,24,25,26].

The usage of nylon fibers in cementitious materials was found to lead to superior performance compared to the usage of PP fibers in terms of compressive strength, splitting tensile strength, modulus of rupture, impact resistance and shrinkage cracks resistance [27]. Recycled PET, PP and HDPE fibers have also been applied to cementitious mortar and found to effectively improve mechanical properties such as toughness, impact load, energy absorption [28,29,30,31,32,33,34,35,36,37].

Currently, the usage of recycled fibers from waste fishing nets is gaining attention among civil engineers, as they have shown the potential to improve the performance of concrete structures [38]; however, only limited research has been conducted. The recycled nylon fibers from waste fishing nets maintains mechanical properties of the virgin fibers [39], and exhibits stability under the high alkaline conditions in concrete [40]. It was found that the addition of recycled nylon fibers from waste fishing nets in cementitious materials increase the tensile strength of mortar under axial load, ductility, and toughness index, and improves flexural capacity in the range of 22–41% [41,42,43]. However, high fiber contents resulted in a reduction of the compressive strength of mortar [40].

This paper investigates the application of polymer cement mortar (PCM) reinforced with recycled nylon (RN) fibers from waste fishing nets for repairing corroded reinforced concrete (RC) beams, with a focus on the restorability of load-carrying capacity of the RC beams exposed to a natural corrosion environment. RC beams were placed in the tidal zone for 14 months to induce steel corrosion, then repairs were carried out by spraying PCM reinforced with RN fiber or manufactured polyethylene (PE) microfiber. The load-carrying capacity, crack openings and final crack formation of the RC beams were extensively investigated to evaluate the effectiveness of the repair.

## 2. Experimental Program

### 2.1. Testing Materials

**Figure 1:**
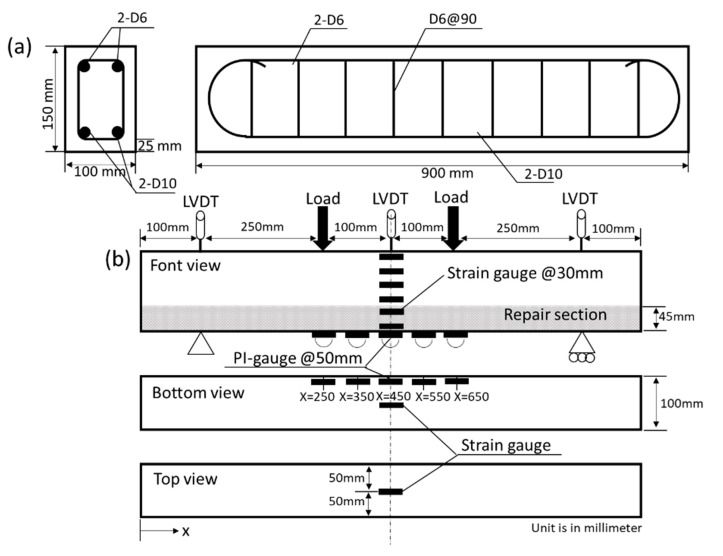
Tested beam: (**a**) geometry and reinforcement details; (**b**) experimental setup.

A total of 12 RC beams measuring 900 mm long, 100 mm wide and 150 mm high were produced. The water-to-cement ratio of the concrete was 0.5, and the design strength was 33 MPa. Galvanized coated steel bars were used for the top (compressive) reinforcement and the stirrups so that the corrosion concentrated on the bottom (tensile) reinforcement. The beams were cured by a wet towel for 7 days, and then placed in the respective environments explained later. Figure 1a shows the geometry and reinforcement details of the RC beams.

**Figure 2:**
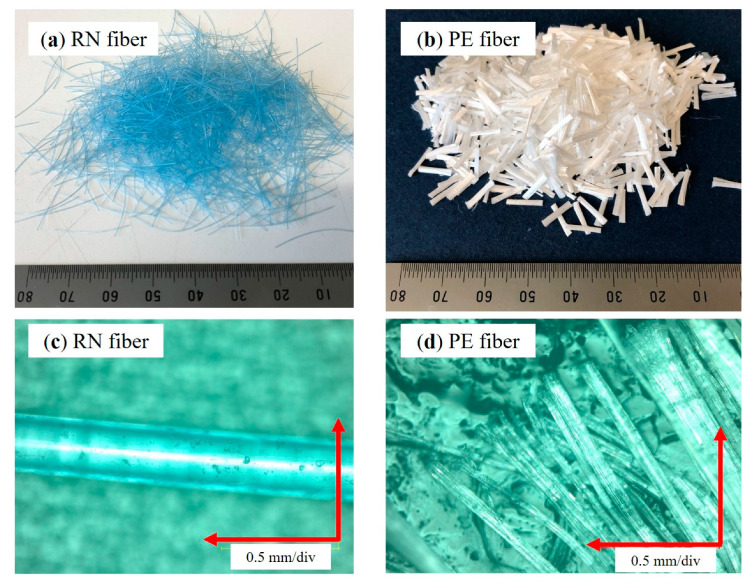
Types of fibers: (**a**) recycled nylon (RN) fiber; (**b**) polyethylene (PE) fiber, and microscope images (×200) of (**c**) RN fiber; (**d**) PE fiber.

The waste fishing nets used in this study were obtained from local fishermen in Hokkaido. The fishing nets were washed by soaking in water, dried, and then manually cut into specified lengths to make RN fiber. Only the straight part of the net was used to prevent the formation of fiber clumps during mixing and to maintain flowability of the fresh mortar. Two types of fiber were used in this study: RN fiber and manufactured PE fibrillated microfiber (Figure 2). PE fibers were produced in the form of bundles, but they separated into individual fibers during mixing with the diameter of 0.01 to 0.1 mm. PCM and PE fibers were supplied from Sumitomo Osaka Cement Co., Ltd., Tokyo, Japan for research purposes.

The uniaxial tensile test was carried out for RN fiber according to the American Society for Testing and Materials (ASTM) C1557 [44], and the properties of the fibers are given in Table 1. Spadea et al. [40] previously confirmed that nylon fiber from waste fishing nets can be safely applied to cementitious materials, with only a slightly drop in tensile strength of around 4% between virgin and used fishing nets.

### 2.2. Exposure and Repair Operations

The tested RC beams were divided into 3 groups: group A beams were stored in the laboratory room for 10 months and then tested; group B beams (normal and upgraded RC beams) were exposed to the tidal zone for 14 months; and group C beams (normal RC beams only) were repaired after the 14-month tidal exposure. The normal RC beam was directly exposed to the tide, and repairs were made at the end of the exposure period. The upgraded RC beams underwent repair operations before starting the exposure, and were not further repaired even after the exposure. The details of the tested beams are listed in Table 2.

For groups B and C beams, steel corrosion was induced naturally by placing the beams in the tidal zone for 14 months. The corrosion progress of the steel rebars was periodically monitored by half-cell potential in accordance with the Japan Society of Civil Engineers (JSCE) E 601 [45].

**Figure 3:**
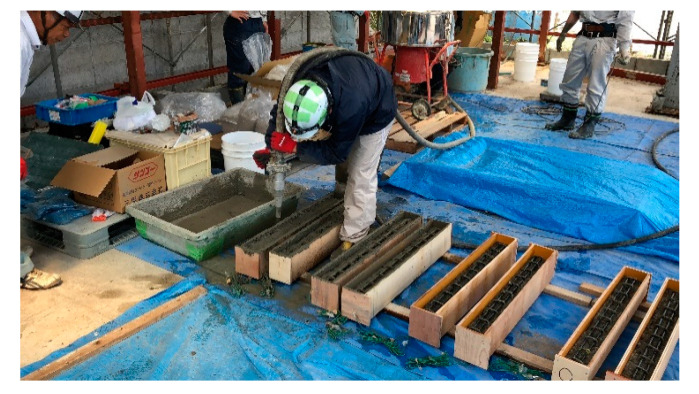
Spraying PCM on the removed section of RC beams.

Repair operations were conducted by removing the bottom concrete cover up to 20 mm over the tensile rebar and subsequently spraying acrylic type PCM reinforced with fibers (Figure 3). Three mixes of PCM were used: PCM without fiber added, or non-fiber, PCM (PCM–NF); PCM reinforced with recycled nylon fiber (PCM–RN); and PCM reinforced with polyethylene fiber (PCM–PE). The water-to-binder ratio of PCM was 0.2, and the fiber fractions by volume for RN and PE fibers were 1.0% and 1.1%, respectively. Mix proportions are indicated in Table 3. It should be noted that the fiber fraction of PE was calculated from the manufacturer in order to simplify the mixing onsite.

PCM was prepared by mixing PCM powder with water for 1 min, followed by air removal agent. Fibers were subsequently added at the designated amount while continuing mixing. The mixing was continued for 2–3 min to ensure uniform fiber distribution and that no fiber clusters have formed. After concluding the mixing, PCM was pumped and sprayed through a nozzle on the removed section of RC beams. PCM specimens were also cast by directly spraying in the 40 mm × 40 mm × 160 mm prism molds and the 50 mm × 100 mm cylinder molds for flexural and compressive tests, respectively.

### 2.3. Loading Tests

Four-point flexural tests, in accordance with Japanese Industrial Standards (JIS) A 1106 [46], were conducted on tested RC beams using 1000 kN type universal testing machine made by Shimadzu Corporation, Tokyo, Japan. Linear variable differential transformers (LVDTs) brand Tokyo Measuring Instruments Laboratory Co., Ltd., Tokyo, Japan with the capacity of 100 mm were used to measure the deflection of the beams at midspan and the end supports. Concrete surface strains were measured using 8 strain gauges with a 30 mm gauge length: 6 gauges attached in the midspan at 30 mm intervals, 1 gauge on the top surface of the midspan, and 1 gauge on the bottom surface. A series of 5 pi-type gauges with 50 mm gauge length were installed at the bottom surface of the beam for measuring crack openings. Figure 1b shows the experimental setup for the tested RC beams.

Flexural strength tests as per JIS R 5201 [47] and the compressive strength tests following JIS A 1108 [48] were conducted on PCM specimens using an autograph-type universal testing machine made by Jacom co., Ltd., Tokyo, Japan with the capacity of 250 kN. These specimens were air cured for 28 days before testing.

## 3. Results and Discussion

### 3.1. Mechanical Behavior of Polymer Cement Mortar (PCM) Specimens

#### 3.1.1. Flowability

Flow diameters of PCM were measured onsite following JIS R 5201 [47], and the results are listed in Table 4. The addition of RN fibers slightly reduced the flow diameter of fresh mortar. For the case of RN fiber in PCM, the reduction in flowability may be negligible; however, further confirmation is needed. A slight reduction of 5–10% in the flowability of fresh mortar was also previously found in the case of the ordinary Portland cement mortar using nylon fiber [40,43]. PCM–PE showed a considerable reduction of 24% in flow diameter compared to PCM–NF. This reduction was likely due to the fibrillated characteristic of the PE fibers, which have a comparatively higher surface area. PE fibers also have lower density than RN fibers; therefore, more fibers are needed to obtain the same volume fraction.

#### 3.1.2. Compressive and Flexural Strengths

The compressive and flexural test results of PCM specimens are summarized in Table 5. The reported values are the average of three samples. The addition of RN fiber resulted in a considerable reduction of both compressive and flexural strengths. However, increases of 24.3% and 39.2% were found for the compressive and flexural strengths, respectively, of PCM–PE. Ozger, et al. [36] suggested that short fiber plays an important role in enhancing the lateral tensile strength of concrete during the load. However, the addition of fibers can increase the voids inside the cement matrix and reduce the modulus of elasticity [12]. It should be noted that the compressive strength tests for PCM-RN were taken from the sawed prism specimen after the bending test.

#### 3.1.3. Failure Behavior

**Figure 4:**
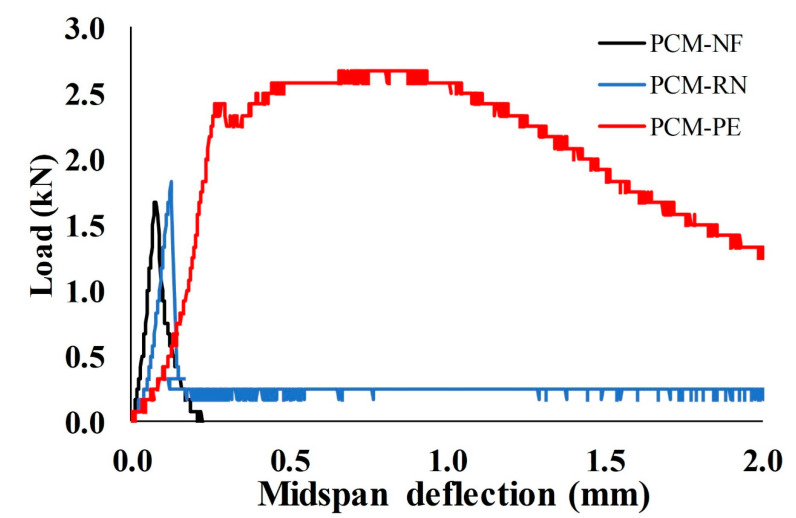
Load-midspan deflection curves of PCM–NF, PCM–RN and PCM–PE.

Three-point flexural tests were performed on 40 mm × 40 mm × 160 mm prism specimens, with the load-midspan deflection curves of the beams shown in Figure 4. PCM–PE showed the highest peak load, followed by PCM–RN and PCM–NF. PCM–NF exhibited a brittle failure mode as the load suddenly decreased to zero, while PCM–PE and PCM–RN retained some load after the yield point. A hardening stage was found for PCM–PE in which the load increased after the peak; therefore, it can be confirmed that PE fibers transferred stresses after the cracks occurred.

For PCM–RN, the load suddenly decreased after the yield point, but still sustained a small residual load of approximately 13.6% of the yield load. Even though the residual load of PCM–RN was lower than that of PCM–PE, RN fiber prevented abrupt failure of the beam. The lower residual load was likely due to weak bonding of the RN fiber, as it has a smooth surface compared to PE fiber. Test results of PCM–RN and PCM–PE correspond to the experiment by Orasutthikul, et al. [43].

**Figure 5:**
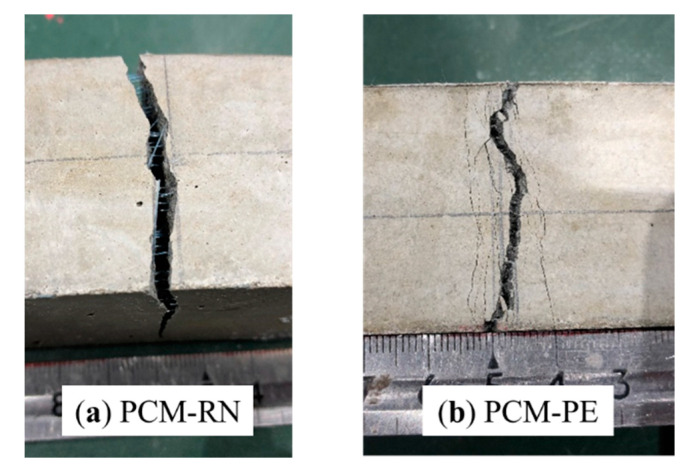
Crack pattern at the bottom surface of the beam: (**a**) PCM–RN; (**b**) PCM–PE.

The final cracks on the bottom surface of the beam after the test are shown in Figure 5. PCM–RN had a wide single crack while PCM–PE had many small cracks. This phenomenon confirmed that PE fiber transfers stresses by dispersing damage from a wide single crack into many small cracks. No breakage of fiber was observed in the case of RN fiber, and RN fibers were pulled out rather than elongated. It is concluded that RN fiber transfers stresses through wide cracks while PE fiber bridges small or micro cracks.

### 3.2. Mechanical Behavior of Reinforced Concrete (RC) Beams

#### 3.2.1. Flexural Capacity

**Figure 6:**
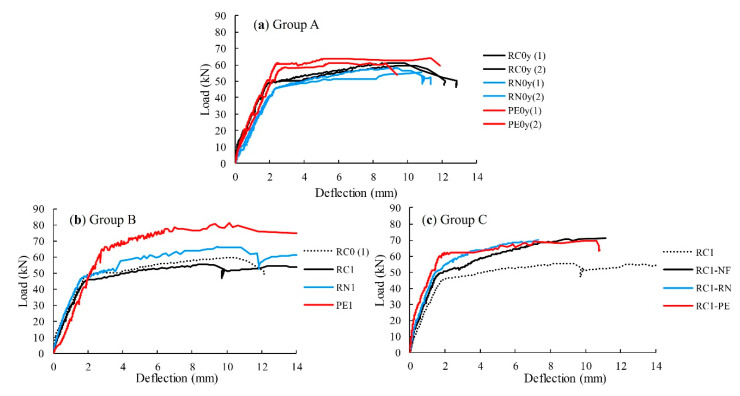
Load-midspan deflection curves of RC beams: (**a**) Group A; (**b**) Group B; and (**c**) Group C.

Results from the four-point flexural tests are summarized in Table 6, and the load-midspan displacement curves are shown in Figure 6. As the flexural capacities of group A beams indicate, the respective two beams having the identical test parameters have an uncertainty of less than 10%. Therefore, the relative trends can be discussed based on the series of tests although only one beam was tested with each set of test parameters for groups B and C beams. For group A, RN0 showed flexural capacity approximately 8% lower than RC0, while PE0 was 7% higher. RN fiber seemed to provide no contribution to the flexural capacity. In addition, the shorter curing period of the beams in group A may be another reason for the decrease in flexural capacity.

For group B, RN1 and PE1 gained 10% and 34% more flexural capacity than RC0, respectively. The increase in flexural capacity of RN1 and PE1 was attributed to the sprayed PCM. The beams sprayed with PCM–RN (RN0 and RN1) tended to express flexural-shear failure mode, likely due to the fact that RN fiber reduces shear capacity by adding voids in the PCM matrix.

For group C, RC1-PE gave highest yield load at 28% higher than that of RC0, but RC1–RN gave a comparable yield load to RC1–NF. The ultimate load of all the beams are similar to each other in the range of 15–17%. PE fibers showed greater contribution to the flexural capacity of the repaired beams than RN fibers; however, flexural capacity of the repaired beams were compensated by spraying PCM. The results of tested RC beams with respect to the sprayed PCM correspond to the mechanical properties of PCM specimens in Section 3.1.2. We conclude from the results that spraying PCM reinforced with RN and PE fibers can restore the load-carrying capacity of the rebar-corroded beams to their original performance.

In general, a section repair is conducted by removal of the deteriorated concrete section and corroded rebars; however, with the usage of reinforced PCM, a section repair can be done without the removal of rebars. The early repair for slightly rebar-corroded beams is intended in this study. Adding fibers was found to compensate the tensile stresses that had been carried by corroded tensile rebars. The beams sprayed with PCM–RN could restore flexural capacity of the corroded RC beams, but were still less effective than PCM–PE.

#### 3.2.2. Strain Distribution

**Figure 7:**
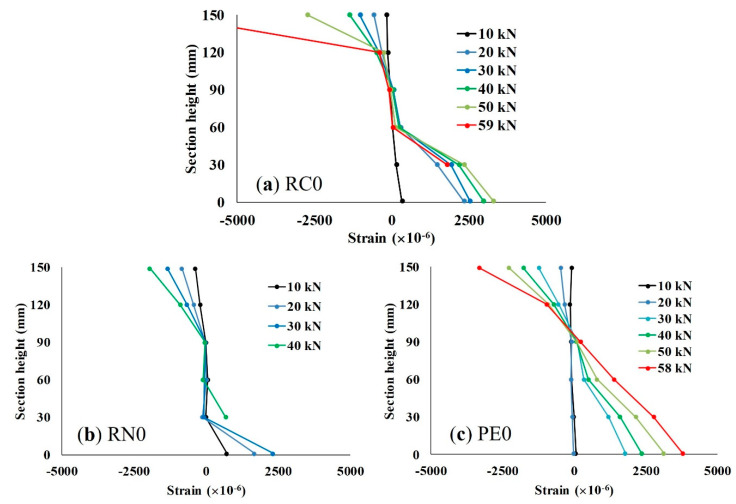
Strain distributions at the midspan of group A beams: (**a**) RC0; (**b**) RN0; and (**c**) PE0.

Concrete strain was measured on the side surfaces of the beams at the midspan with the spacing of 30 mm. Strain distributions of the RC beams in group A are shown in Figure 7. PE0 showed almost linear strain distribution while RC0 and RN0 showed unbalanced strain distributions. For PE0, strain seems to increase linearly as the load increased, indicating that no separation occurred between PCM and the substrate concrete. The same tendency of uniform distribution of strain is also observed in groups B and C. The addition of fiber—especially PE—seems to help distribute strain throughout the beam. However, in some cases, cracks propagated outside the measurement area, and the strain gauge could only measure strain before it broke. Therefore, it is difficult to explain the actual phenomenon of the strain during the loading.

#### 3.2.3. Crack Formation

**Figure 8:**
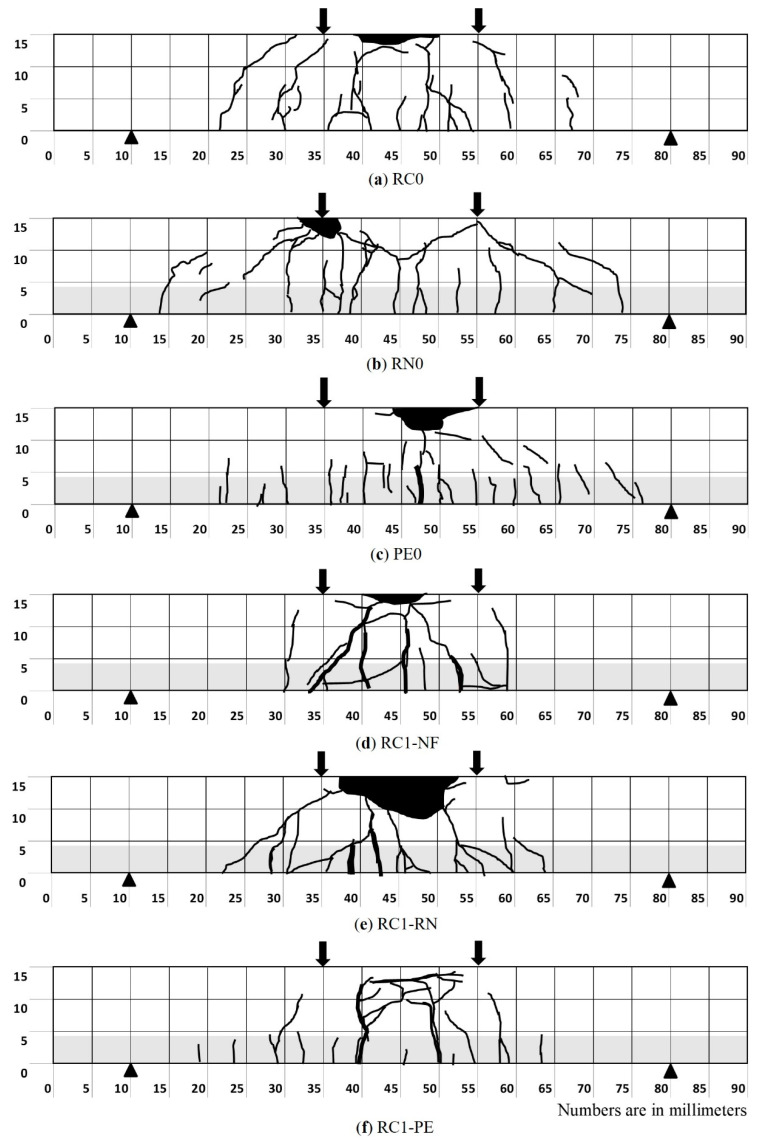
Final crack formation of the tested beams. (**a**) RC0; (**b**) RN0; (**c**) PE0; (**d**) RC1–NF; (**e**) RC1–RN; (**f**) RC1–PE.

The sides of the test beams were divided into many small grids for observing crack formation. Cracks were drawn manually on the beam just after the experiment, then plotted on a spreadsheet. Although only the cracks on the front side of the beams are reported, it was observed that the crack patterns on the back side of the beams were similar. Figure 8 shows the final crack formations on the side surface of the beams after the four-point bending test.

**Figure 9:**
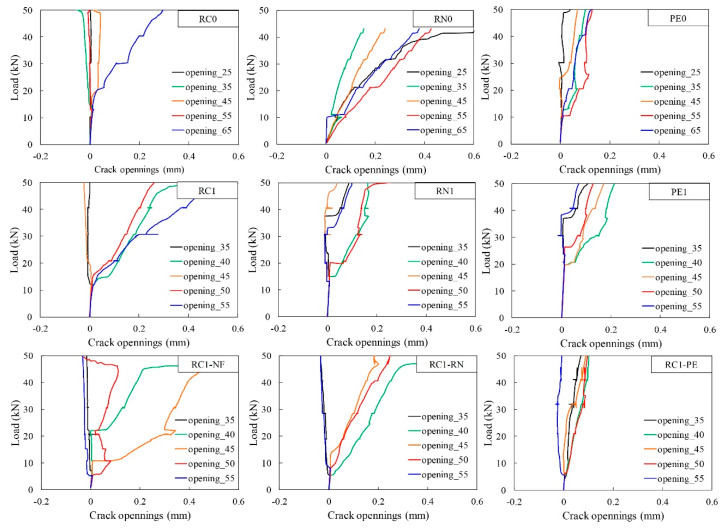
Crack openings at the bottom surface of the tested beams.

Crack openings on the bottom surface of the beams were also measured at the location of 25, 35, 45, 55, and 65 cm from the end of the beam, with the results plotted in Figure 9. It should be noted that for RC1–NF, RC1–RN and RC1–PE, the locations of the pi–type gauges were changed to 35, 40, 45, 50, and 55 cm because cracks tended to concentrate at the midspan of the beam.

The beams sprayed with PCM–RN and PCM–PE fibers showed more distributed cracks comparing to the normal RC beam. Especially for PE0 and RC1–PE, many small cracks were observed. For RN0 and RC1–RN, cracks were widely distributed and spread towards the support, rather than concentrated near the midspan of the beam. In contrast, cracks were observed to be most concentrated at the midspan for RC0 and RC1–NF. Adding fiber seems to facilitate stress transfer through cracks; therefore, fibers prevent serious damage from wider cracks by distributing into many small cracks. However, it should be noted that cracks formed in the outer side of the gauge-covered areas cannot be measured, which makes it difficult to evaluate the total increment rate of the crack opening.

#### 3.2.4. Rebar Mass Loss

All the beams were demolished after the flexural tests, and the tensile rebars were carefully collected. The rebars were washed using diammonium hydrogen citrate solution, and the mass of the remaining tensile rebar was measured. The percent mass losses of each tensile rebar are listed in Table 6. All the exposed beams had an average mass loss of 0.5% to 0.8% with RC1 exhibiting a slightly higher loss of 1.8%. The mass losses of RN1 and PE1 are slightly lower than that of RC1, likely due to the effect of fibers that reduce permeability and mitigate steel corrosion; however, further investigation is needed. Rebar mass loss confirmed that all exposed beams had almost the same level of corrosion.

## 4. Conclusions

This experimental study on PCM reinforced with RN and PE fiber for the repair of corroded RC beams reveals several findings. Results confirmed that recycled nylon fibers from waste fishing nets have great potential for the effective repair and upgrading RC structures, as they showed performance comparable to the manufactured PE fibers. As such, application of recycled fiber for civil engineering is a possible way of utilizing waste fishing nets. From this study, the following conclusions were drawn:

1. The addition of RN seems to have no noticeable effect on the flowability of fresh PCM. However, the addition of PE fiber results in a reduction of flowability of PCM up to 23.7% due to the geometry and surface property of PE fiber.
2. The addition of RN fiber results in a reduction of compressive strength and flexural capacity of PCM. In contrast, the addition of PE fiber increases the compressive and flexural strengths of PCM up to 24% and 39%, respectively.
3. The addition of RN and PE fibers enables the PCM to sustain more flexural loads after the peak. PCM-RN retains a stable post-peak load of approximately 13.6% of the yield load, while the use of PCM-PE led to an increase in the load after the yield point. The addition of RN short fiber helps prevent abrupt failure of the beams; however, its flexural strength is inferior to those of PE.
4. RN and PE fibers can be used as a reinforcement material for the repair of lightly corroded RC beams. Spraying reinforced PCM can compensate the flexural capacity that deteriorated due to the corrosion of tensile rebar; however, the effectiveness of the RN fibers is inferior to that of PE fibers.
5. Adding fibers helps distribute stresses throughout the beam under the bending load. RN fiber helps transfer stresses through wide cracks and spreads the cracks toward the support of the beams. PE fiber prevents severe damage of the beams by distributing damage from a wide crack to many small cracks.
6. The rate of crack openings is reduced in the RC beams repaired by PCM reinforced with fiber. The beams that were repaired with PCM-PE exhibit the lowest crack opening and the rate of crack openings, followed by PCM–RN.

The recycled nylon fibers from waste fishing nets tested in this study have been proven effective for reinforcing cementitious materials, and for repairing lightly corroded RC beams. It must be noted that, even though the effectiveness of using RN fiber is slightly inferior to the manufactured PE fiber, the use of recycled fiber promotes waste utilization from the ocean.

Further studies are still necessary in terms of pre-treatment, quality control of the recycled fiber, and durability against longer exposure. At the experimental scale, RN fibers were manually cut by hand, which is time consuming and not suitable for real applications. Processing machines need to be developed for mass production of RN fibers. Pre-treatment of fiber is also necessary to improve bond behavior between fibers and the cement substrate. Finally, factors affecting the durability are yet to be investigated, such as permeability, pore distribution and the chloride ion resistivity of the fiber-reinforced mortar.

## Figures and Tables

**Table 1:** Properties of the fibers.

Fiber	Diameter (mm)	Length (mm)	Density (g/cm^3^)	Tensile Strength (MPa)
Recycled nylon (RN)	0.24	20	1.13	440
Polyethylene (PE)	0.01–0.1	9	1.05	n/a

**Table 2:** Name and conditions of the tested RC beams.

Specimen Name		Condition	Tidal Exposure	Sprayed Material
Group A	RC0	Normal RC	No	-
	RN0	Upgraded RC	No	PCM–RN
	PE0	Upgraded RC	No	PCM–PE
Group B	RC1	Normal RC	14 months	-
	RN1	Upgraded RC	14 months	PCM–RN
	PE1	Upgraded RC	14 months	PCM–PE
Group C	RC1–NF	Normal RC	14 months	PCM–NF
	RC1–RN	Normal RC	14 months	PCM–RN
	RC1–PE	Normal RC	14 months	PCM–PE

**Table 3:** Mix proportion of PCM (in 25 kg of PCM).

Mix	Water (kg)	Fiber (g)
PCM–NF	4.93	-
PCM–RN	4.93	0.164
PCM–PE	4.93	0.160

**Table 4:** Flow diameters.

Specimen Name	Flow Diameter (mm)	%ΔFlow Diameter
PCM–NF	165	-
PCM–RN	164	−0.7
PCM–PE	126	−23.7

**Table 5:** Compressive strength tests and flexural strength tests results.

Specimen Name	Compressive Strength Tests			Flexural Strength Tests			
	*f’_c_* (MPa)	*SD*	%Δ*f’_c_*	*P_cr_* (kN)	*f_b_* (MPa)	*SD*	%Δ*f_b_*
PCM–NF	32.7	3.65	-	1.69	4.77	0.14	-
PCM–RN	24.0 ^1^	3.53	−31.4	1.56	4.37	0.72	−8.4
PCM–PE	43.5	1.41	24.3	2.36	6.64	0.82	39.2

Note: *f’_c_* is the compressive strength, *SD* is the standard deviation, %Δ*f’_c_* is the percent difference in compressive strength compared to PCM–NF, *P_cr_* is the maximum load, *f_b_* is the flexural strength, and %Δ*f_b_* is the percent difference in flexural strength compared to PCM–NF. ^1^ Obtained with 40 mm cube specimens taken out from the beam.

**Table 6:** Four-point flexural test results.

Specimen Name		*P_y_* (kN)	*P_u_* (kN)	%Δ*P_y_*	%Δ*P_u_*	Failure Type	Mass Loss of Each Tensile Bar (%)	
Group A	RC0 (1)	49.1	59.6	-	-	Flexural	-	
	RC0 (2)	48.1	60.9	-	-	Flexural	-	
	RN0 (1)	41.4	55.4	−14.8	−8.0	Flexural–shear	-	
	RN0 (2)	45.1	58.0	−7.2	−3.7	Flexural–shear	-	
	PE0 (1)	58.5	61.0	20.4	1.2	Flexural	-	
	PE0 (2)	60.7	64.2	24.9	6.6	Flexural–shear	-	
Group B	RC1	45.2	56.2	−7.0	−6.7	Flexural	0.9	1.8
	RN1	48.3	66.5	−0.6	10.4	Flexural–shear	0.8	0.7
	PE1	64.6	81.1	32.9	34.6	Flexural	0.6	0.7
Group C	RC1–NF	49.1	70.8	1.0	17.5	Flexural–shear	0.6	0.5
	RC1–RN	50.2	69.9	3.3	16.0	Flexural	0.6	0.5
	RC1–PE	62.0	69.7	27.6	15.7	Flexural	0.8	0.9

Note: *P_y_* is the yield load and *P_u_* is the ultimate load and %Δ*P_y_* and %Δ*P_u_* are the percent difference in *P_y_* and *P_u_* compared to the average of RC0 (1) and RC0 (2), respectively.
